# Cigarettes-induced acute eosinophilic pneumonia: a case report

**DOI:** 10.1186/1757-1626-1-414

**Published:** 2008-12-23

**Authors:** Leenhapong Navaravong, Kitsada Wudhikarn, John J Marini

**Affiliations:** 1 Medicine Education Office, MMC 284 Mayo, 8284, 420 Delaware Street SE, Minneapolis, Minnesota, 55455, USA; 2 Pulmonary and Critical Care Medicine, Regions Hospital, 640 Jackson St, Room 3571, St Paul, Minnesota, 55101, USA

## Abstract

**Background:**

Idiopathic acute eosinophilic pneumonia is a rare disease. It presents with acute febrile illness, respiratory insufficiency, pulmonary infiltration and high eosinophil levels in Bronchoalveolar lavage fluid. Pathogenesis is not well understood, but may relate to the exposure to exogenous substances.

**Case presentation:**

We present a case of 20-year-old man, who developed idiopathic acute eosinophilic pneumonia after smoking cigarettes and required intubation with mechanical ventilation. His symptoms resolved quickly after corticosteroids therapy.

**Conclusion:**

Acute eosinophilic pneumonia should be considered in the differential diagnosis of a patient with acute febrile respiratory illness, diffuse bilateral pulmonary infiltrates and recent modification in smoking habit.

## Case presentation

A 20-year-old Caucasian male, was transferred to our medical intensive care unit (MICU) with fever, progressive dyspnea and worsening chest radiograph, despite initial antibiotics. Patient denied history of using illicit drugs, food or drug allergies, and recent camping or wild animal contact. He was had occasionally smoked cigarettes, had abstained for three years before presentation. Two days prior to this admission, he had immense stress and smoked a whole pack of cigarettes within a few hours. Later, he gradually developed fever with chills, non-productive cough, and dyspnea within the same day of smoking. He presented to another hospital and was admitted for Community Acquired Pneumonia. Ceftriaxone and azithromycin were given empirically without improvement. According to worsening of symptoms and pulmonary infiltrates despite antibiotics, patient was transferred to MICU for further management.

On physical examination, he was in marked respiratory distress, temperature of 100.8°F, blood pressure of 127/65 mmHg, pulse 110 beats per minute. Crackles without wheezes were audible in both lungs. No pedal edema or Jugular venous distension was noted. There were no skin lesions, lymphadenopathy, joint swelling or erythema. With severe respiratory distress, the decision was made to intubate patient.

**Figure 1:**
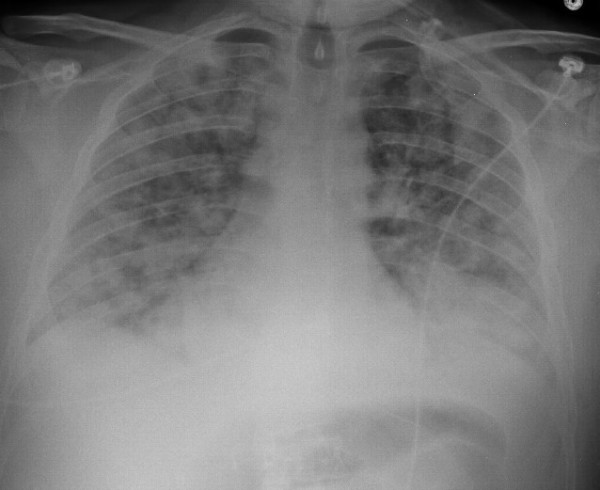
**Chest radiograph showed bilateral pulmonary infiltrates**.

**Figure 2:**
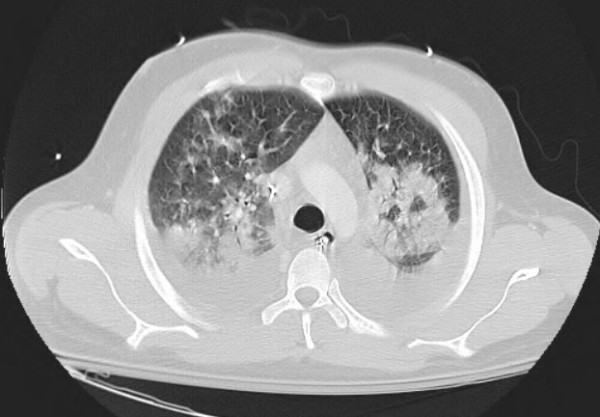
**Computed tomography of the chest showed diffuse alveolar infiltration bilaterally with pleural effusions**.

**Figure 3:**
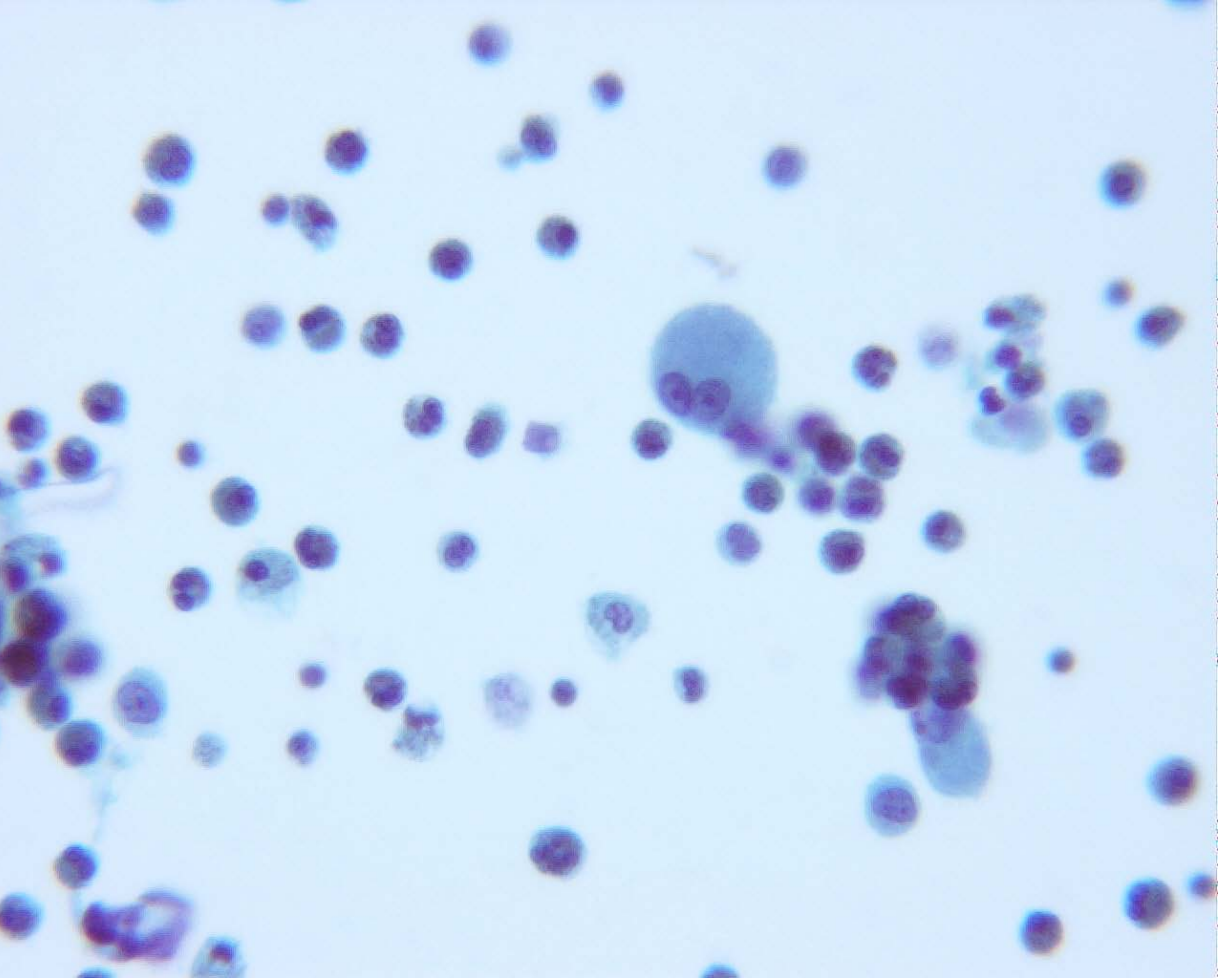
**Bronchoalveolar lavage fluid demonstrated more than 25% eosinophils**.

The laboratory tests revealed WBC count of 16,600/μL, polymorphonuclear leukocytes, 88%; lymphocytes, 5%; monocytes, 4%; eosinophils, 3%. His blood chemistries were normal. The chest radiograph showed diffuse infiltrates bilaterally (fig 1). Transthoracic echocardiogram revealed normal systolic function and pulmonary pressures. Computed tomography (CT) of the chest showed diffuse bilateral alveolar infiltration with bilateral pleural effusions (fig 2). Broad spectrum antibiotics were started for possibility of severe pneumonia. His blood cultures, sputum cultures, and urine legionella antigen were negative. With no response to antibiotics, bronchoscopy was performed with bronchoalveolar lavage (BAL). Bronchoscopy demonstrated inflamed mucosa in both upper lungs with thick secretions in both lower lungs. BAL fluid showed nucleated cells 2080/μL with eosinophils, 73%; lymphocytes, 15% (fig 3). There was no evidence of cytomegalovirus, bacteria, fungus, Pneumocystis jiroveci, parasites and malignancy. These results supported a diagnosis of idiopathic acute eosinophilic pneumonia.

**Figure 4:**
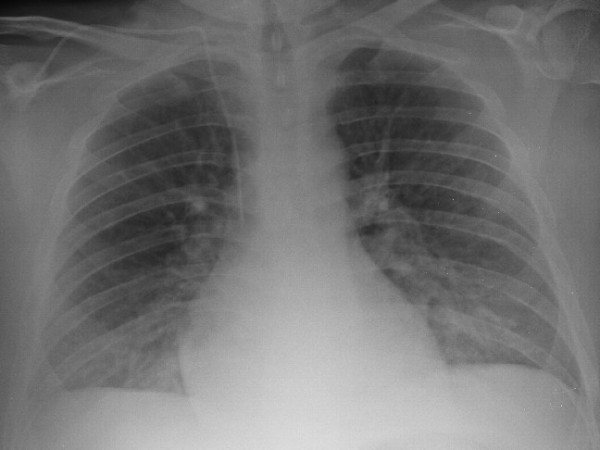
**Chest radiograph showed significant improvement after treatment with systemic corticosteroids**.

Patient was started on methylprednisolone intravenously, and antibiotics were discontinued. Within two days after initiation of steroids, patient was successfully extubated. Repeat Chest radiograph showed significant radiographic improvement (Fig 4). He was discharged to home, after five days in the hospital, with a course of prednisone tapering for 14 days.

## Discussion

Idiopathic Acute Eosinophilic Pneumonia (IAEP) is a rare disorder with estimated incidence of 9.1 per 100,000 person years [1]. IAEP is characterized by the following: (1) acute onset of febrile respiratory illness with duration less than one month, usually less than seven days; (2) hypoxemic respiratory failure; (3) diffuse bilateral infiltrates on chest radiographs; (4) pulmonary eosinophilia with more than 25% eosinophils in BAL fluid; (5) absence of infection, medications or other known causes of eosniophilic lung disease [2,3].

Multiple presumptive causative agents have been reported; outdoor activities [2,4], cigarette smoking [2,4-6], and airborne sand or dust exposure including the World Trade Center dust [7].

The relationship between cigarette smoking and IAEP has been reported in many literatures, mostly in individual with recent cigarette smoking [1,2,4-6]. Rechallenge of smoking can cause recurrence of disease [6], while some patients develop tolerance when continue smoking [8]. Furthermore, recent published study by Uchiyama et al [9], suggested that not only beginning to smoke, but also restarting or increasing amounts, are associated with IAEP.

Chest radiograph shows bilateral pulmonary infiltrates, mixed alveolar and reticular opacities up to ground glass opacities [2]. CT chest shows ground-glass opacities, consolidation, nodular opacities and pleural effusion [2]. BAL is the major diagnostic modality for IAEP. The eosinophil count greater than 25% is the key part of diagnosis [1,2,4].

The differential diagnoses of idiopathic acute eosinophilic pneumonia are broad. Fungal and parasites are the main infectious etiologies that need to be excluded. Some medications have also been associated with eosinophilic pneumonia (see appendix 1).

Corticosteroids are the main treatment of this disease. The response is usually with in 24–48 hours without relapse following withdrawal of corticosteroids. Spontaneous improvement has also been reported. Prognosis is excellence in almost all cases. Death is exceptionally rare [1]. After resolution of respiratory failure, prednisone will be continued at a dose of 40–60 mg/day orally for 2–4 weeks then tapering over 4 to 12 weeks until complete the course of treatment. Alternative diagnosis should be considered if there is no response to high-dose corticosteroid therapy [10].

## Abbreviations

MICU: Medical Intensive Care Unit; CT: Computed tomography; BAL: Bronchoalveolar lavage; IAEP: Idiopathic acute eosinophilic pneumonia.

## Competing interests

The authors declare that they have no competing interests.

## Authors' contributions

LN and KW wrote the manuscript and performed the literature search; JJM reviewed the manuscript for intellectual content.

## Consent

Written informed consent was obtained from the patient for publication of this case report and accompanying images. A copy of the written consent is available for review by the Editor-in-Chief of this journal.

## Appendix

### Appendix 1

Differential diagnoses for idiopathic acute eosinophilic pneumonia [10]

• Parasitic disease

• Fungal infection

• Drug-induced pulmonary eosinophilia

∘ Antibiotics (nitrofurantoin, dapsone, clarithromycin, minocycline, ethambutol, isoniazid)

∘ Chemotherapeutic agents (methotrexate, bleomycin, fludarabine)

∘ Sulfasalazine

∘ Nonsteroidal antiinflammatory drugs

∘ Hematopoietic stem cell transplantation

∘ Amiodarone

∘ Captopril

∘ Phenytoin

• Rheumatoid arthritis

• AIDS
